# Mental distress among young adults – gender differences in the role of social support

**DOI:** 10.1186/s12889-021-12109-5

**Published:** 2021-11-24

**Authors:** Rune Johansen, Mari Nicholls Espetvedt, Heidi Lyshol, Jocelyne Clench-Aas, Ingri Myklestad

**Affiliations:** 1 Norwegian Institute of Public Health, P.O. Box 222, Skøyen, N-0213 Oslo, Norway; 2 Vestfold and Telemark County Council, P.O. Box 2844, N-3702 Skien, Norway

**Keywords:** Mental distress, Social support, Depression, Mental health, Anxiety, Sense of coherence

## Abstract

**Background:**

The aim of the present study was to examine to what extent observed gender differences in mental health are associated with the protective factors social support, sense of coherence and participation in regular physical activity and more generally, engagement in organized or unorganized activity with other people.

**Methods:**

This study was based upon a cross-sectional regional health survey in Norway, conducted during the winter of 2015–2016, in three southern counties; Aust-Agder, Vest-Agder and Vestfold. The study focused on young adults, comparing three age groups; 18–24 years old (*n* = 624), 25–31 (*n* = 582), and 32–38 years old (*n* = 795).

**Results:**

Sense of coherence was strongly associated with low mental distress in all age groups and for both genders, while the association between low social support and mental distress was significant for young women only. Regular physical activity was not positively associated with low mental distress when sense of coherence and social support were included in the analysis.

**Conclusion:**

Social support appears to have a stronger role as a protective factor for mental distress among young women, compared to young men and older persons. This has implications for health promoting activities that target young women. Sense of coherence showed a strong association with low mental distress scores for all ages studied.

**Supplementary Information:**

The online version contains supplementary material available at 10.1186/s12889-021-12109-5.

## Background

Mental health problems among young people have become an important issue within public health. Several European studies conducted in recent years have found high and increasing prevalence of symptoms of mental distress in young people, in particular among young women [1–3], with levels in the 25–40% range among young women, and 15–30% range among young men [4–6]. A large study from Norway in 2019 showed a prevalence of symptoms of mental distress of 33% among 18–19 year old girls and 14% among boys of the same age [7].

Although the levels of mental distress may vary due to study design and choice of instrument, and between countries or regions, the main message seems clear; mental problems are common, and the gender gap in mental distress is most pronounced among young people.

The review study by Bor et al. [1], based upon 19 studies, suggests that recent cohorts of adolescent girls are experiencing increasing levels of internalizing symptoms compared to previous cohorts. The same review shows that during the first decade of this century, a substantial increase in the levels of mental distress among young women was reported, although in later years levels seem to have stabilized somewhat. In Norway there has been a similar trend. A large study among adolescents 15–19 years shows that among boys the level of high symptoms of mental distress was stable at 6% in the years 2011–2016, while among girls high symptoms of mental distress was 15.9% in 2011 and increased to 19.7% in 2016 [8].

Because of the apparent frequent occurrence of mental health issues among young people, it is interesting to explore the processes behind the problem for a greater understanding.

### The role of protective factors for mental health

Mental distress results from a complex interplay of biological, social, environmental and behavioural factors. Influences from childhood events, genetic and psychosocial factors are commonly assumed to be important causal factors for mental distress [9, 10]. Negative factors for mental health and mental health problems have been well studied and are diverse. Examples are factors that have a major impact on daily life, such as employment status, economic hardship and various other adverse life events [3–5]. In addition, lifestyle factors such as physical inactivity, smoking, being underweight, and risk consumption of alcohol have been associated with symptoms of poor mental health [3–5, 11, 12]. On the other side are protective factors for mental health, which often receive less public attention. Apart from increasing an individual’s quality of life and wellbeing, protective factors may be of crucial importance for mobilising resources and sustaining mental health in the face of negative life events. In this paper, the protective factors are emphasized.

An important psychosocial factor for young people is social support. Slightly differing, but related definitions exist for social support. In a theoretical framework social support has been described with two important dimensions, a structural dimension and a functional dimension [13, 14]. The structural dimension includes social network size and frequency of social interaction, while the functional dimension includes emotional support (receiving love and empathy) and instrumental support, including for example practical help with daily tasks or economic help. Alternative conceptual models concerned with how social support affect psychological health exists, including perspectives on how type of social support meets the specific needs of the recipient [15, 16].

The processes that link social support to health and well-being at an individual level are complex. A full consideration of this is beyond the scope of this study. Still, on a population level patterns of associations between mental well-being and from whom there is perceived support also give some clues. For example, previous studies have shown that social support from family and friends is an important protective factor for mental distress among both young people and adults [3–5, 11, 17–21]. A large population-based health study from Norway, including approximately 9000 Norwegian adolescents (13–19 years old), found that social support from friends was the most important protective factor against psychological distress among adolescents and young people [22].

The literature on social support demonstrates that both adolescents and adult women report higher levels of perceived social support from friends and family compared to men [23, 24]. Although gender differences in various aspects of social support seem clear, evidence for gender differences in the association between social support and mental health is mixed. A review study from 2016 of young people [25], showed that social support was a significant protective variable against depression among men and women. Some studies have however showed that low social support was related to higher levels of depressive symptoms to a greater extent among women, than men [26, 27]. The results from the literature show that different social relationships and forms of social support have different impact on mental health among men and women [28, 29]. For young women, friendships with other young women had the strongest social support effect on depressive symptoms, whereas for young men, the support of their teacher had the strongest effect [28]. Myklestad et al. [20] found that a social unstructured leisure activity such as “hanging out with friends during leisure time”, was more important for mental health among young women adolescents, compared to men. However, structured leisure activities, such as joining an athletic club, was more important for adolescent men’s mental health. For adult populations, a study by Wareham found that emotional support from family and friends might be more beneficial for mental health for women than men [29]. Overall, inconsistencies in findings across source of support and characteristics of the support recipient suggest that important questions remain about when and to whom social support confers positive benefits.

Previous studies have also shown that organized leisure activities were beneficial to youth mental health because they connected young people to more positive peer support, introduced specific social, physical, and intellectual skills, provided a setting for personal exploration, and offered practice dealing with challenges [30–32]. Participating in organized activities among adults has also been attached to positive experiences and life satisfaction [33–35]. Both unorganized and organized leisure activities has been shown to be related to social support, which may play a critical role in people’s ability to cope with stress [36, 37].

In the field of health promotion, the salutogenic model of Antonovsky gives insight into the connection between stress, coping and health [38]. Social support would in the salutogenic model be placed among what is termed general resistance resources, that is, individual or environmental resources that can be used to counter the stressors of everyday lives [39]. Within other frameworks social support is also described as a form of social capital [2], which indicates a similar function as a protective element. At the core of the salutogenic model is the product, or overarching concept, sense of coherence (SOC). SOC includes the three elements comprehensibility, manageability and meaningfulness, and it is concerned with how people’s perceptions of these elements will influence successful stress or tension management in daily life and challenges. It is well established that low SOC are related to reduced mental health and quality of life [40]. Although often focused on the individual level, SOC is also applicable to different system levels, as within groups (family- and otherwise), or on organizational and societal level [41].

### Aims of the paper

The aim of this study was to examine how a selection of protective factors were associated with mental distress. Especially of interest were protective factors that may explain gender and age differences in mental distress. Such knowledge may in turn be of importance for finding ways to reverse the recent negative trends observed for mental distress. Protective factors included were social support, sense of coherence, participation in regular physical activity, and participation in organized and unorganized activities.

## Methods

### The sample

The data used for analysis were a subsample from a cross-sectional health survey conducted in three southern Norwegian counties; Vestfold, Aust-Agder and Vest-Agder, from November 2015 to February 2016. The complete study sample was drawn randomly from the adult population; aged 18 years and older. Individuals living at institutions were excluded. In order to maximize the response rate, the respondents could choose between three sampling methods: a postal questionnaire, an electronic web-based questionnaire, or interview by phone. Invited respondents got the opportunity to answer by post or web before they alternatively were contacted by phone.

The gross sample for the survey was 22,700. The overall response rate was 42.7, 40.2% among men and 45.2% among women, giving a net sample of 9692, including 4551 men and 5141 women. 7122 (73.5%) responded by post, 1821 (18.8%) by web, and 749 (7.7%) by phone.

The subsample used for analysis included young adults 18 to 38 years old, in total 2001 individuals. The subsample was further divided into three age groups for analysis, 18–24, 25–31 and 32–38 years old. Individuals in the youngest age group was of particular interest in this study and included 261 men and 363 women, in total 624 individuals. The response rate in this age group was noticeably lower than for the whole study, only 23.4% as a group, 19.3% among men and 27.8% among women. Table 1 below summarizes the total number of participants in the three age groups and split by gender.

**Table 1:** Study sample with respect to age and gender

		Men	Women	Total
**Age groups**	**18–24**	261	363	624
	**25–31**	242	340	582
	**32–38**	350	445	795
	**Total**	853	1148	2001

### Measures

#### Mental distress

The outcome variable in this study was the five-item version of the 25-point Hopkins’ Symptom Check List scale (HSCL-5) [42]. The symptoms of mental distress measured by HSCL-5 are: (1) Nervousness or shakiness inside, (2) Feeling fearful, (3) Feeling hopeless about the future, (4) Feeling blue/sad, and (5) Worrying too much about things.

Each of these five items was measured on a four-category scale: «not at all», «a little», «quite a bit», and «extremely»; with scores from 1 to 4. The cut-off used was an average value of 2.0 [43].

#### Social support

*Social support* was measured using the Oslo 3-item social support scale [44, 45]. This instrument consists of three questions which broadly measures aspects of both structural and instrumental social support; (1) How many people are so close to you that you can count on them if you have serious personal problems? Answering categories “none”, “1–2”, “3–5” and “more than 5”, (2) How much concern and interest do people show in what you are doing? Answering categories “a lot of concern and interest”, “some concern and interest”, “neither great nor slight concern and interest”, “little concern and interest”, “no concern and interest”, (3) How easy is it to get practical help from neighbours if you should need it? Answering categories “very easy”, “easy”, “neither easy nor difficult”, “difficult”, “very difficult”. The sum score of these three questions, indicating the level of social support from lowest to highest, ranges from 3 to 14.

A cut-off of 10.0 was used for low and high social support. The Oslo-3 item social support scale has been operationalized into three broad categories of social support: 3–8 poor social support, 9–11 moderate social support and 12–14 strong social support [44, 46]. The cut-off was therefore based on a combination of recommended cut-off points [46], and consideration of ensuring sufficient statistical units in each group, and due to descriptive statistics indicating a natural divide when plotted against HSCL-scores.

#### Sense of coherence

The study also included a three-item short version of Antonovsky’s 29-item measure “Sense of coherence” (SOC), and captures the three main dimensions of SOC; comprehensibility, manageability, and meaningfulness [47–50]. The wordings of the items were: “(1) Do you usually see solutions to problems and difficulties that other people find hopeless? (2) Do you usually feel that your daily life is meaningful and satisfying? (3) Do you usually feel that the things that happen to you in your daily life are hard to understand?”. The three response categories were: “Yes, usually”, “Yes, sometimes”, and “No”. These response categories are scored from 0 to 2, with the scoring being reversed for question number three, in order to align the answers in the same direction. Thus, the sum score is between 0 and 6. In order to dichotomize, a cut-off point at 2 and below was chosen to represent high SOC, this cut-off point was recommended by Lundberg & Peck [49].

#### Social and physical activities

Lifestyle variables known to be potentially protective to mental health were selected. This included physical activity, defined broadly to include a variety of activities, from typical exercise activities, biking to and from work and school, to walking trips and gardening. Physical activity was measured using an integral scale with six categories, from 1 to 6 with the response categories: “never”, “less than once a week”,” once a week”, “2-3 times a week”, ”4–5 times a week” and “nearly every day”. Two other questions asked about frequency of engagement in organized and unorganized activities, were scored on an integral scale between 1 and 4 (from “never” to “daily”), with the response categories: “never”, “1-3 times a month”, “once a week” and “daily”. Organized activities were defined as any activity or voluntary work carried out connected to for example a sports club, a political society, a religious society, choir or similar. Unorganized activity was exemplified in the questionnaire as meeting friends, exercise trip with friends, colleagues or others. A dichotomous version of these three variables was used in the study, grouped into participation less than once per week (value = 0) versus participation once a week or more frequently (value = 1).

### Treating missing values

The individuals who did not respond to one or more of the questions in the study were treated as missing and were not included in the analyses. No imputation of the missing data was done. The exception was HSCL, where standard procedure is that when one item is missing, the average response of all the other questions should be used, with two or more missing items, the individuals are excluded from the analysis. The total sample size was 2001, and the number of missing values was for most variables between 29 (physical activity) and 63 (HSCL). The exceptions were sense of coherence and social support, where 319 and 322, respectively, were missing. This seems to be due to a design weakness of the postal questionnaire. Excluding missing values yielded a net sample of 1643 in the total model.

### Statistical analyses

In line with the aim of the study, the focus of the logistic regression analysis was mainly concerned with protective factors, namely participation in organized and unorganized activities, participation in regular physical activity, social support, and SOC.

To reveal potential gender and age differences in factors associated with mental health, six stratified groups were created and analysed separately. The age and gender groups used for comparison have been summarized in Table 1.

**Fig. 1:**
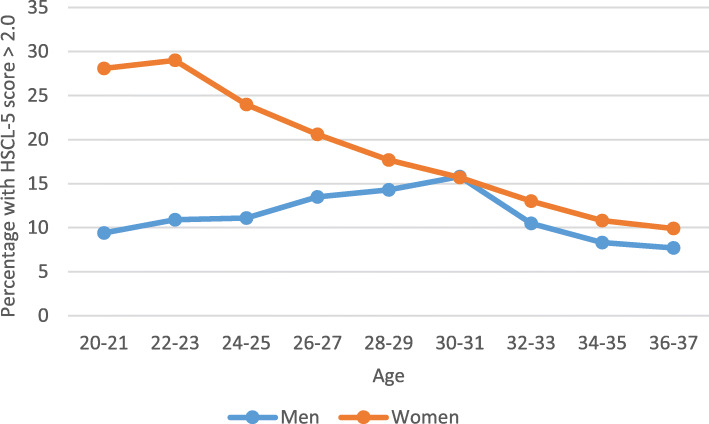
Mental distress, fraction having HSCL-5 score > 2.0, among young people; 18–38 years old, both genders. Two-year averages

Initially, various descriptive and explorative analyses were carried out. This included the prevalence of mental distress (HSCL-5 score > 2), plotted in a trend line for men and women, based on two-year age groups and including ages 20 to 37 (see Fig. 1). Further patterns for mean HSCL-5 scores in relation to high social support or low social support were investigated, and likewise frequencies of dichotomized covariates of interest, both including the six stratification groups based on gender and age.

Correlation analysis of the outcome variable, mental distress, and covariates of interest was carried out, both to give initial information on strength of correlation and to check for excessive multicollinearity between potential covariates for the regression models. Non-normality of the data and categorical variables made it appropriate to use the Spearman’s *ρ* as a correlation measure.

Both multivariable logistic and linear regressions were carried out, including interaction terms, thus viewing the linear model mainly as a supplement to better study interactions and model fit. The main focus was differences in mental health with respect to protective factors across gender and age. Explorative analyses indicated gender and age differences in the association between social support and mental health. Therefore, the only interaction terms considered were then involving social support, age and gender: gender x social support, age x social support, and age x gender x social support. In the linear model, the continuous version of all the variables were used. The total model included 1643 individuals, excluding those with missing values.

To acquire more detailed knowledge about gender and age differences in factors associated with mental distress (mean HSCL-5 score > 2), a multivariable logistic regression model was fitted separately to the data for men and women in the three age groups, 18–24, 25–31 and 32–38 years, thus yielding six different models.

Analyses were carried out using IBM SPSS version 27.0.

## Results

### Descriptives

Based on the two-year averages (Fig. 1), the level of mental distress observed is higher among young women than young men. The level of mental distress among women appears to reach its maximum in the early twenties, with a maximum level of about 30%, while among men, the level appears to culminate at about 15%, at an age of about 30 years.

Table 2 shows the prevalence of proposed protective factors of mental distress by gender and age group. In early adulthood it appears to be more common to participate frequently in unorganized activities as opposed to organized activities, but with a notable decreased prevalence with increasing age. Around 70% of both men and women aged 18–24 participate in unorganized activities weekly or more often. Most participants reported high social support, in total 79.1%. Low social support was highest for women in the youngest age group, at 26.5%. The prevalence of high SOC appear to increase with increasing age and was in the range of 69% for women 18–24 years old to 88.9% for men 32–38 years old. Overall the proportion of men with high SOC is slightly higher than that of women, 82.1% and 79.1% respectively.

**Table 2:** The prevalence of the covariates included in the study, presented by gender in the three age groups 18–24 and 25–31 and 32–38 years old and all ages combined, and men and women, separate and combined

Dichotomized covariates	Age group	Men (%)	Women (%)	Both genders (%)
Organized activities, participation weekly or more often	18–24	30.2	28.4	29.2
	25–31	20.4	20.2	20.3
	32–38	30.4	35.6	33.3
	All ages	27.5*	28.7**	28.2**
Unorganized activities, participation weekly or more often	18–24	69.3	71.9	70.8
	25–31	52.9	58.3	56.0
	32–38	46.8	49.4	48.3
	All ages	55.4**	59.1**	57.5**
Regular physical activity, carried out weekly or more often	18–24	62.2	64.4	63.5
	25–31	60.7	67.1	64.4
	32–38	61.4	67.5	64.8
	All ages	61.5	66.4	64.3^**§**^
High social support (scale 3–14, cut-off point at 10)	18–24	78.0	73.5	75.3
	25–31	76.6	82.7	80.1^**§§**^
	32–38	81.4	81.3	81.4
	All ages	79.0	79.2*	79.1
High sense of coherence (scale 0–6, cut-off point at 2.0)	18–24	74.1	69.0	71.1
	25–31	80.8	81.9	81.5
	32–38	88.9	85.6	87.1
	All ages	82.1**	79.1**	80.4**

**Note:** Chi-square test was used to test significant differences between genders and three age groups for the covariates in the study. Significant results are marked with */§ *p* < 0.05, **/^**§§**^ *p* < 0.01, ^**§**^denotes gender difference, and * denotes difference between age groups. The symbols are placed on the prevalences for combined ages and genders

The differences between age groups were significant (*p* < 0.01) for both unorganized and organized activities, and for both genders (see Table 2). The gender difference was significant (*p* < 0.05) for physical activity. The gender difference was also significant for social support in the age group 25–31 years. For women there was a significant difference between age groups (*p* < 0.05) for social support. Finally, there was a significant difference between age groups (*p* < 0.01) for sense of coherence, and for both genders.

**Fig. 2:**
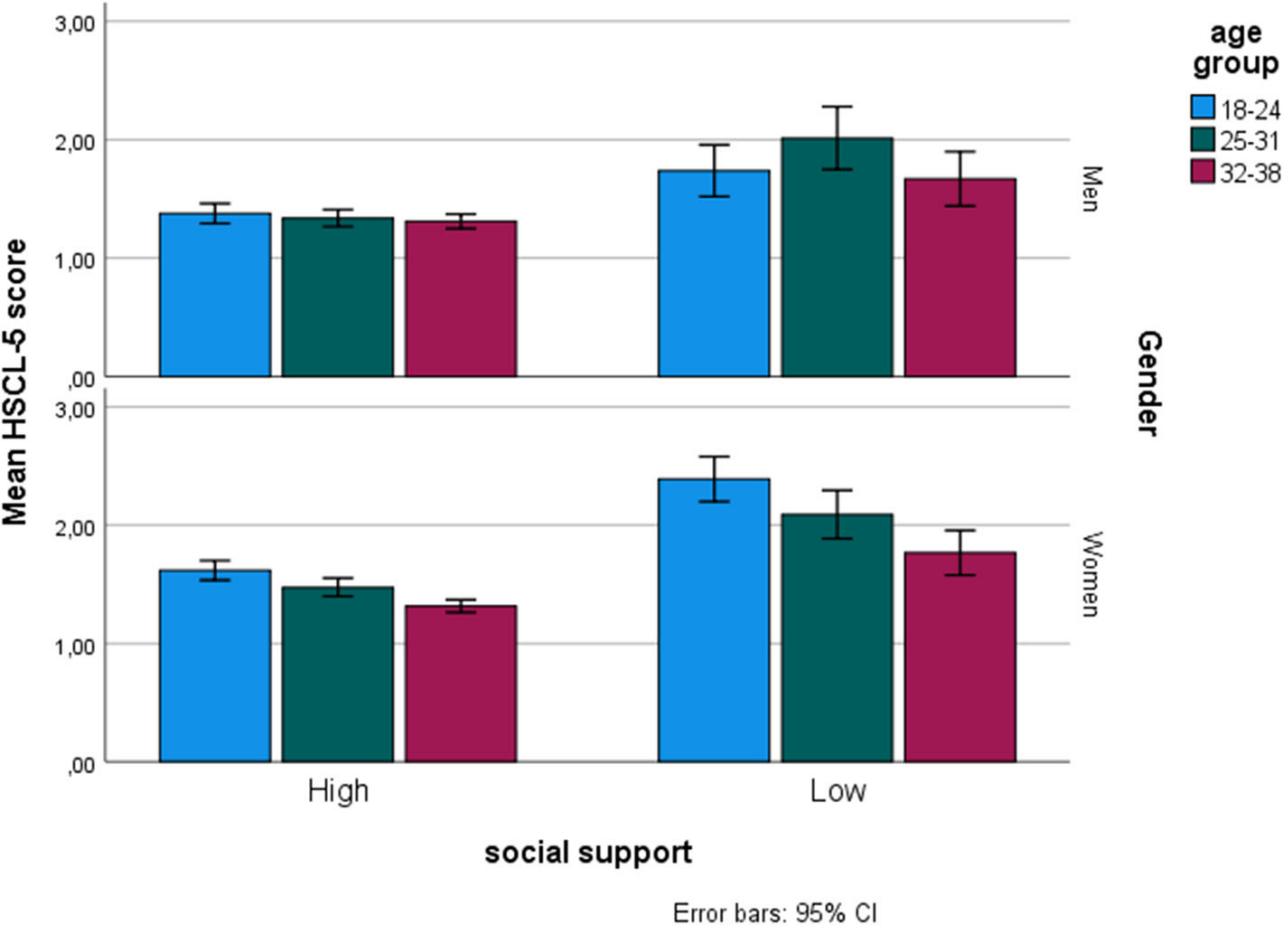
Mean HSCL-5 score versus high or low social support, in three different age groups of men and women separately. Error bars indicate 95% confidence intervals

The mean HSCL-5 score shows an obvious pattern of lower scores when social support is high and higher HSCL-5 scores, indicating mental distress of above 2.0, with low levels of social support, in all age groups and for both men and women (Fig. 2). For women in the age group 18–24 years old the mean HSCL-5 among those with low social support (*N* = 80) was 2.39 (95% CI = 2.20–2.58), indicating on average that most young women experiencing low social support also fell into the category of mental distress. For the sake of comparison, the mean HSCL score among those young women experiencing high social support (*N* = 222) was 1.62 (95% CI = 1.53–1.70), as shown in Fig. 2 below.

### Correlations

Table 3 shows that four of the five predictors included in the model had univariate significant association with young (18–24 years old) women’s mental health. These were participating in unorganized activities, participating in physical activity, social support and SOC. For young men, however, only three predictors were significantly associated with mental distress (HSCL-5): participating in unorganized activity, social support and SOC. Among 25–31-year-old women, participation in organized activities rather than regular physical activity was significantly associated with mental health (Table 4), while among 32–38 year-old men it was only social support and SOC that were associated with the HSCL-5 score (Table 5).

**Table 3:** Correlation matrix using Spearman’s ***ρ***; age group 18–24 years; men above the diagonal, women below the diagonal

	HSCL	SOCIAL SUPPORT	SENSE OF COHERENCE	UNORG. ACTIVITIES	ORGANIZED ACTIVITIES	PHYSICAL ACTIVITY
HSCL		0.23**	−0.35**	− 0.15*	− 0.07	−0.09
SOCIAL SUPPORT	0.41**		−0.24**	−0.20**	− 0.03	−0.24**
SENSE OF COHERENCE	−0.40**	−0.42**		0.22**	0.06	0.07
UNORG. ACTIVITIES	−0.19**	−0.38**	0.24**		0.24**	0.27**
ORGANIZED ACTIVITIES	−0.06	−0.19**	0.02	0.13*		0.21**
PHYSICAL ACTIVITY	−0.11*	−0.15**	0.14*	0.16**	0.17**

**Table 4:** Correlation matrix using Spearman’s ***ρ***; age group 25–31 years; men above the diagonal, women below the diagonal

	HSCL	SOCIAL SUPPORT	SENSE OF COHERENCE	UNORG. ACTIVITIES	ORGANIZED ACTIVITIES	PHYSICAL ACTIVITY
HSCL		0.35**	−0.43**	−0.13*	−0.07	− 0.06
SOCIAL SUPPORT	0.39**		−0.43**	−0.11	− 0.09	−0.09
SENSE OF COHERENCE	−0.48**	−0.40**		0.12	0.02	0.15*
UNORG. ACTIVITIES	−0.16**	−0.31**	0.23**		0.22**	0.27**
ORGANIZED ACTIVITIES	−0.17**	−0.08	0.05	0.17**		0.08
PHYSICAL ACTIVITY	−0.07	−0.04	0.07	0.20**	0.05

**Table 5:** Correlation matrix using Spearman’s ***ρ***; age group 32–38 years; men above the diagonal, women below the diagonal

	HSCL	SOCIAL SUPPORT	SENSE OF COHERENCE	UNORG. ACTIVITIES	ORGANIZED ACTIVITIES	PHYSICAL ACTIVITY
HSCL		0.21**	−0.36**	−0.06	−0.05	0.01
SOCIAL SUPPORT	0.29**		−0.31**	−0.14*	− 0.21**	−0.07
SENSE OF COHERENCE	−0.51**	−0.33**		0.07	0.07	0.11
UNORG. ACTIVITIES	−0.13**	−0.21**	0.11*		0.29**	0.18**
ORGANIZED ACTIVITIES	−0.08	−0.13*	0.02	0.25**		0.17**
PHYSICAL ACTIVITY	−0.04	−0.13*	0.05	0.31**	0.14**	

### Logistic regression

After initial correlation analysis and model testing, including considering effect sizes, five covariates remained in the final six stratified models. These were participation in organized and unorganized activities, physical activity, social support, and SOC.

Table 6 shows the result of the multivariable binary logistic regression analysis, with dichotomized HSCL-5 score as dependent variable, and including interaction terms gender x social support, age x social support, and gender x age x social support. The table shows that there was a significant interaction between gender x age x social support. This suggested that stratified analyses on both age and gender were needed in order to reveal how this effect affects the different age groups and the two genders.

**Table 6:** Multivariable binary logistic regression analyses. Mental health (HSCL-5) with respect to various covariates, including interaction terms between social support, age and gender. Significant association in bold face. *n* = 1643

Variable	OR	Sig	CI (95%)
Participation in unorganized activities	0.988	*p* = 0.944	0.701–1.391
Participation in organized activities	**0.606**	***p*** = 0.014*	**0.406–0.903**
Physical activity	0.923	*p* = 0.640	0.660–1.292
Social support	**0.263**	***p*** < 0.01**	**0.131–0.528**
Sense of coherence	**0.139**	***p*** < 0.01**	**0.100–0.195**
Gender	0.601	*p* = 0.167	0.292–1.236
Age	0.606	*p* = 0.175	0.294–1.242
Gender x social support	0.410	*p* = 0.078	0.152–1.105
Age x social support	0.988	*p* = 0.977	0.423–2.309
Gender x age x social support	**0.338**	***p*** < 0.01**	**0.149–0.587**

Note: **p < 0.01 and *p < 0.05

Linear analyses using continuous versions of the same covariates as in Table 6 were also performed, see appendix 1. The results from the linear regression analyses showed that the interaction between gender x age x social support was significant. The explained variance (adjusted *R*^2^) was 0.389.

Tables 7, 8, and 9, one for each age group, show the results of the multivariable binary logistic regression analyses stratified on age and gender. The three variables involving participation in activities are not significantly associated with mental health, except for women in the middle age group (25–31 years), and then with regard to participation in organized activities, OR = 0.18 (95% CI = 0.05–0.60). The odds ratio gives an indication of how protective the significantly associated covariates may be for mental distress. Social support shows a highly significant association among 18–24 and 25–31-year-old women, OR = 0.22 (95% CI = 0.11–0.42) and 0.22 (95% CI = 0.09–0.52) respectively, and a significant but smaller association for women 32–38 years old, OR = 0.39 (95% CI = 0.16–0.96), and men 25–31 years old, OR = 0.37 (95% CI = 0.14–0.98). SOC is a highly significant predictor for both genders in all age groups. In addition, it appears that SOC increases as a reliable predictor of less mental distress with increasing age, as shown by narrower confidence intervals with increasing age. The 95% confidence intervals for SOC for women 18–24 years old and that for women 32–38 years old do not overlap.

**Table 7:** Multivariate binary logistic regression analyses. Mental health (HSCL-5) with respect to five covariates. Men and women 18–24 years old

	Men		Women	
Variable	OR	CI (95%)	OR	CI (95%)
Participation in unorganized activities	0.93	0.33–2.63	0.93	0.48–1.82
Participation in organized activities	0.62	0.18–2.13	0.90	0.45–1.80
Physical activity	0.81	0.28–2.33	0.89	0.48–1.64
Social support	0.46	0.16–1.32	0.22**	**0.11–0.42**
Sense of coherence	0.15**	**0.05–0.41**	0.29**	**0.16–0.54**

Note: **p < 0.01 and *p < 0.05

**Table 8:** Multivariate binary logistic regression analyses. Mental health (HSCL-5) with respect to five covariates. Men and women 25–31 years old

	Men		Women	
Variable	OR	CI (95%)	OR	CI (95%)
Participation in unorganized activities	0.56	0.22–1.43	0.76	0.35–1.65
Participation in organized activities	0.75	0.21–2.63	0.18**	**0.05–0.60**
Physical activity	0.99	0.39–2.47	0.53	0.25–1.12
Social support	0.37*	**0.14–0.98**	0.22**	**0.09–0.52**
Sense of coherence	0.15**	**0.06–0.39**	0.11**	**0.05–0.26**

Note: **p < 0.01 and *p < 0.05

**Table 9:** Multivariate binary logistic regression analyses. Mental health (HSCL-5) with respect to five covariates. Men and women 32–38 years old

	Men		Women	
Variable	OR	CI (95%)	OR	CI (95%)
Participation in unorganized activities	0.93	0.36–2.44	0.88	0.33–2.38
Participation in organized activities	0.96	0.32–2.94	0.48	0.17–1.35
Physical activity	0.53	0.19–1.49	0.77	0.28–2.08
Social support	0.44	0.17–1.36	0.39*	**0.16–0.96**
Sense of coherence	0.09**	**0.03–0.26**	0.06**	**0.02–0.14**

Note: **p < 0.01 and *p < 0.05

Table 10 gives an overview of the sample sizes and model fits in the six stratified models. Considering the Cox & Snell and Nagelkerke indicators suggests that the chosen number of covariates gives an acceptable model fit.

**Table 10:** Sample size and explained variance (Cox & Snell and Nagelkerke) for the model using five covariates, for all six combinations of age groups and gender

		Men			Women		
		18–24	25–31	32–38	18–24	25–31	32–38
Model, 5 covariates	Sample size	200	203	275	287	263	334
	Cox & Snell	0.11	0.17	0.10	0.20	0.24	0.19
	Nagelkerke	0.22	0.29	0.23	0.28	0.37	0.38

## Discussion

The results from the present study show that the level of mental distress is higher among 18–24-year-old women, compared to both men in the same age group, and older age groups up to 38 years old of both genders. The gender difference is confirmed by earlier studies [2–4, 27] but differences are not equally clear for age. Still, possibly the most interesting finding of this study was the apparently stronger association social support had with young women’s mental health compared to that of young men.

### Prevalence of mental distress and gender

There is no clear and simple explanation why mental distress in most surveys shows an apparently higher prevalence in women compared to men. Other authors have pointed towards several different reasons. For example, Charney pointed out that young men have more difficulties in acknowledging their mental health problems and tend to mask this by acting out their difficulties instead [51]. This may result in more externalising disorders, such as antisocial personality disorders and substance abuse or dependence among young men [51, 52]. Young women, on the other hand, report more internalizing disorders such as depression [2] and anxiety [52–54].

Gender difference may also be related to the socially defined roles of women and men, which in many societies exposes them to gender-specific stressors [2]. Young women suffer for example more from stressors which involve interpersonal social relationships [55], experience more restricted gender roles and body dissatisfaction [56, 57], and experience more family violence, abuse and school pressure [58, 59], which all have been associated with a greater likelihood of mental health problems [2].

In understanding the gender difference in prevalence of mental distress another perspective can be found in the field concerned with psychobiology, studying the body’s physiological stress mechanisms [51]. For example, it has been suggested that physiological stress activation occurs partly due to different kinds of stressors in men and women [60], and one study found higher levels of the stress hormone norepinephrine in women compared to men [61].

In addition, another consideration in understanding the gender difference in prevalence of mental distress, is the age pattern. In this study, it appears that the prevalence for mental distress reaches its highest level approximately 8–10 years earlier in women than in men. Although not presenting exactly the same age groups as in our study, Molarius et al. [4] include a figure that also points towards a gender difference in the age at which mental distress shows a peak in prevalence. This is a complex field, which encompasses looking at differences in brain development for men and women [62] and age undergoing puberty, related to cognitive development [63, 64].

### Social support and gender

The study revealed that social support had a stronger association with young women’s mental health compared to that of young men’s mental health. Social support was directly associated with mental health among both young men (18–24 years old) and young women before controlling for the other protective factors. The results of the multivariate analyses however, showed that social support was still associated with mental health problems among young women after controlling for other protective variables. Among young men however, social support was no longer significantly associated with mental health. This suggests that social support may play a key role in the observed difference in mental distress between young women and young men. Our results concerning social support are partly consistent with earlier studies. Earlier studies have showed mixed results regarding whether social support influence differently on mental distress for young men and women. The results from a large review study, showed that social support was a significant protective variable for depression among both young men and women [25]. Another study by Luo et al. from 2017 [28] showed that different social relationships had different impact on mental health among men and women. For girls, the social support of same-sex friendships had the strongest effect on depressive symptoms, whereas for boys, teacher–student relationships did. Results from a longitudinal Australian study that investigated young people’s mental health over a period of 13 years, showed that the mental health of women appeared to benefit slightly more from higher levels of social support from friends and family than men [26].

A possible reason why social support seem to be a more important protective factor for mental distress among young women compared to men may be found in previous studies that have shown that young women experience more stress in interpersonal social relationships and are more likely to become depressed as a consequence of peer and family stress exposure compared to young men, [55, 65–67].

A gender difference in statistical significance on the effect of social support on mental health was also present in the oldest group, 32–38 years old. However, in this group there were almost the same odds ratios for both men and women, and the confidence intervals strongly overlapped.

### Physical activity

Regular physical activity alone was significantly associated with mental health problems among young women (18–24 years). Physical activity was however not a significant predictor among young women when controlled for other protective factors such as social support, SOC, and participation in organized and unorganized activities. Thus, it seems like social support and SOC was the most important protective factors for mental health among young women, and that the social aspects of physical activity and the way activities gave meaning in daily life, were more important for young women’s mental health. Previous studies are partly consistent with these findings [68–71]. In addition, previous research has showed that participating in social activities, like team sports, is more important for mental health than physical activity per se [72–76].

### Sense of coherence

The results from the present study show that SOC was highly associated with mental health in both men and women in all included age groups. This likely emphasises how the perception that everyday life events are comprehensible, meaningful and possible to master (manageability), have a positive impact on mental health, regardless of gender among those 18 to 38 years old. This is consistent with the studies of Antonovsky [47], and in later studies investigating the correlation between quality of life and SOC [38]. Antonovsky argued that SOC is a cross-cultural concept and that it is human to seek understanding, to cope with daily challenges, and to seek meaning in the various aspects of life. The consistent strong association SOC had with mental health, for both genders and all ages, indicates that any health promoting activity or intervention, regardless of specific target group and main focus, should pay careful attention to SOC. Super et al. [38] suggested two closely interlinked processes, based on an exploration of the salutogenic model, that may need to be included in health promotion activities with the aim to strengthen SOC. In short these two are the process of empowering people to identify appropriate resources to deal with everyday stressors and the second process is focused on facilitating reflection to increase understanding of the stressor they are facing, to better identify available resources and to give a feeling that dealing with stressors can be meaningful. In Super et al. [38] the authors refer to a selection of intervention studies that has been successful in increasing SOC levels. They argue that for example the study by Kähönen et al. [77] contained group interventions that targeted both empowerment and reflection in employees with severe burnout symptoms.

### Limitations of the study

It is important to stress that these analyses are based on cross-sectional survey data. Thus, it is neither possible to decide whether there really is causation, nor is it possible to point out the direction of the suggested causation. Young women may experience mental problems because of a lack of social support. On the other hand, it is also possible that mental problems influence social support, e.g. that young women with mental problems withdraw from other people or are excluded from social groups. Although the data are not suitable for examining causation, a cross-sectional study may reveal significant gender differences when predictors for mental problems are concerned.

Commonly, 9.0 has been recommended as a cut-off point for social support [44]. In general, and as far as possible, the choice of cut-off should be based on the statistical properties of the variable under consideration. In the present material, there were very few young men (*n* = 17) scoring below 9.0. In order to meet this challenge, a higher cut-off level of 10.0 was used for both genders. Comparing the social support scores with those of HSCL, there is a clear gradient. Lower social support is strongly associated with high HSCL-scores, and descriptive statistics show an apparent divide when the social support score is 9 or 10. For those with social support score of 9 or lower, the fraction having HSCL-scores above 2.0 is well above 20%, while among those with social support score of 10 or higher, less than 15% had a high HSCL-score.

Another limitation of the study is the low response rate of 23% among the youngest age group (18–24 years), particularly among young men. This may have led to selection bias.

The subsample used for this study was not checked for selection bias, but the complete material from the cross-sectional health survey showed an increasing response for older age up to 80 years old, and noticeably, but less than for age, a higher proportion of women and of those with completed higher education participating. A certain amount of selection bias may therefore be assumed, but it is difficult to determine how this may have impacted the findings of this study.

Another limitation of this study was the number of protective factors available for analysis. The questionnaire was primarily designed with the aim of giving sufficient overview of the population health of the participating counties, and at the same time ensuring a short questionnaire.

Sociodemographic covariates, like education, income and employment are important in the general adult population, but not necessarily correct or representative of socioeconomic status for young adults. The majority of young Norwegians under 25 have neither finished their education, nor have they acquired high-paying jobs yet. Many of our study participants are therefore too young to offer good data on education, employment and income, and these variables were not included in the study. Family background, like parents’ education and income, may be more relevant and important background factors, but such data were not available.

The sum score of social support used in this study is a robust tool for measuring social support. However, the “help from neighbour”- dimension of the social support-scale might be less relevant for young people today. Young people communicate with their social network through social media to a large extent and may be less concerned with social support from their physical neighbours. The social support measure might be most effective when matched to the individual’s preferences. However, the data used in this study were not intended for studying such details on a population level. In future work on social support, this should therefore be considered.

### Implications of the study

Bearing in mind that the material for this study originated from a general public health survey, without defining a specific setting or target group other than age, the findings may still be interesting and relevant to research in different fields and for various groups of young adults and practical settings. From a public administration and public policy making perspective there is a desire to both gain a greater understanding of and simultaneously achieve a reduction in the apparent high prevalence of mental distress in the general population, particularly among young women. With this perspective in mind, two areas of focus are suggested. Firstly, a further exploration of whether there are specific aspects of social support that especially need attention among young adults. For example, Jiang et al. [78] found an age difference in type of social support seeking comparing older adults (age 60+) and young adults (age 18–25). Compared to older adults, young adults were found to seek more explicit social support, that is, emotional comfort that involves disclosure and discussion of problems and the request for assistance. After such an identification the next step would be to gain understanding of opportunities on how to strengthen those specific aspects of social support.

Secondly, the findings add support to existing, and hopefully will inspire to new, health promotion activities, that include a focus on increasing SOC, and activities that strengthen social support as an available resistance resource. In the literature there appear to be more examples of mental health promoting interventions including specific at risk or treatment groups [79, 80], but the principles of empowerment and findings on influence on social capital and social cohesion should still be relevant for the general population. Health promoting activities with a salutogenic perspective may benefit from careful attention to including elements of both empowerment and reflection [38], and an exploration of strengthening SOC at different system levels [81]. Interventions may also include attention to how the environmental conditions for health may be changed, and then specifically regarding changes that may increase mental health through access to social support. This for example by increasing opportunities for social interactions in the physical environment or through organized activities. Commers et al. [82] proposed an analytical instrument to use for strategies to influence various aspects of physical and social environments for health. Included in this instrument is the role of the public health professional when empowering groups or individuals to gain ability to undertake suitable environmental actions.

Finally, it may be important to emphasize that most of young adult women 18–24 years old, are in an educational setting, and as such this is possibly where suitable changes or interventions for increased social support may be most relevant, although local communities may also play a role.

## Conclusion

This study found a notable stronger association between mental distress and social support in regression models for young women compared to young men. Thus, social support appears to have a somewhat different role as a protective factor for mental distress among women, particularly for those 18–24 years old, compared to other groups studied. This might be important to bear in mind for health promoting activities that target young women. Further investigations of whether certain factors underlying perceived social support are of higher significance compared to others, and in that case how to best strengthen these, may lead to useful knowledge with an aim to reduce the high prevalence of mental distress.

In addition, elements underlying the concept SOC (sense of coherence) are of interest in all ages studied, as high scores for SOC showed a strong association with low mental distress scores. Engagement in physical activity was not positively associated with low mental distress once SOC and social support were included in regression models, possibly indicating that activity per se is less important than the perceived meaningfulness and social aspects involved.

## Supplementary Information

**Additional file 1.** Appendix 1. Linear regression

## Data Availability

Data used in this study are available upon request from the Norwegian Institute of Public Health (NIPH) and after permission from the county councils of Vestfold, Aust-Agder and Vest-Agder. Researchers can apply for access to the survey data here: https://www.fhi.no/en/more/access-to-data/ . Extra restrictions apply to the availability of the data with variables from national registries that require permission from the registry owners and the Norwegian Data Protection Authority.

## Abbreviations

SOC: Sense of Coherence
HSCL: Hopkins Symptom Check List
OR: Odds Ratio
CI: Confidence Interval
NAV: Norwegian Labour and Welfare Administration
WEB: World Wide Web Internet
