# Supplementary material for: Mental distress among young adults – gender differences in the role of social support
Source: BMC Public Health. 2021 Nov 24;21:2152. doi: 10.1186/s12889-021-12109-5 (PMC8611886; doi:10.1186/s12889-021-12109-5)
Supplement: Supplementary file 1 — Additional file 1. Appendix 1. Linear regression [file 12889_2021_12109_MOESM1_ESM.docx]

**Appendix 1.**

**Linear regression**

The results of the multivariable linear regression analysis, including age and gender as covariates, and with interaction terms, yielded further insight into statistical patterns of the available data (see Table A1). Table A1 shows the linear multiple regression analysis with the covariates age and gender and the same five covariates as in the logistic regression analysis. Table A1 shows that social support and sense of coherence (SOC) have a significant association with mental health (HSCL-5), and physical activity, participation in organized and unorganized activity were not statistically significant.

As the main focus of the present study is differences in mental health with respect to social support across gender and age, we have only considered interaction terms involving social support, age and gender; gender x social support, age x social support, and age x gender x social support. The total model included 1643 individuals, excluding those with missing values. The results from Table A1 show that there are significant interaction effects between social support and age, social support and gender, and the three-way interaction term: social support x age x gender. The three-way interaction term shows that there is a significant difference of gender and age on the association between social support and mental health.

## **Table A1;** Multivariable linear regression analyses. Mental health (HSCL-5) with respect to various covariates, including interaction terms between social support, age and gender. Significant association in bold face. Explained variance adjusted $R^{2}$-score=0.389, n=1643.

| **Variables** | **B** | ***p*** | **CI (95%)** | **Standardized**$\beta$ |
| --- | --- | --- | --- | --- |
| Participation in unorganized activities | 0.001 | 0.978 | [-0.036, +0.037] | 0.001 |
| Participation in organized activities | -0.001 | 0.923 | [-0.032, +0.029] | -0.002 |
| Physical activity | 0.005 | 0.627 | [-0.015, +0.024] | 0.010 |
| Social support | **-0.156** | **p<0.01**** | **[-0.238, -0.074]** | **-0.464** |
| Sense of coherence | **0.268** | **p<0.01**** | **[0.244, 0.291]** | **0.512** |
| Gender | 0.174 | 0.241 | [-0.117, +0.464] | 0.131 |
| Age | -0.021 | 0.069 | [-0.043, +0.002] | -0.194 |
| Gender x social support | **0.041** | **0.020*** | **[0.007, 0.075]** | **0.403** |
| Age x social support | **0.004** | **p<0.01**** | **[0.002, 0.006]** | **0.559** |
| Gender x age x social support | **-0.002** | **p<0.01**** | **[-0.002, -0.001]** | **-0.510** |

Note: **p< 0.01 and *p< 0.05.

We have also run linear analyses stratified on age and gender, in the same manner as with the logistic models (tables not shown). The effect of social support on men’s mental health in the youngest age group (18-24 years) was partially significant. The standardized beta for men in this age group was about half ($\beta=-0.133$) compared to the similar coefficient for women ($\beta=-0.256$), suggesting that the effect for women was much stronger, thus confirming the results from the stratified logistic models.
